# Machine-Learning Predictive Tool for the Individualized Prediction of Outcomes of Hematopoietic Cell Transplantation for Sickle Cell Disease: Registry-Based Study

**DOI:** 10.2196/64519

**Published:** 2025-09-15

**Authors:** Rajagopal Subramaniam Chandrasekar, Michael Kane, Lakshmanan Krishnamurti

**Affiliations:** 1 Section of Pediatric Hematology/Oncology/BMT, Yale School of Medicine, 2073 A, LMP Builidng 330 Cedar Streeet, New Haven, CT, United States, 1 412-612-4761; 2 School of Data Science and AI, Indian Institute of Technology, Madras, Chennai, India; 3 Department of Lymphoma-Myeloma, The University of Texas MD Anderson Cancer Center, Houston, TX, United States

**Keywords:** sickle cell disease, SCD, prediction algorithms, hematopoietic stem cell transplantation, machine learning, ML, predictive tool, prediction, hematopoietic cell transplantation, HCT, hematopoietic cell, registry-based study, clinical decision-making, prediction model, clinical outcomes, gene therapy, shared decision-making

## Abstract

**Background:**

Disease-modifying therapies ameliorate disease severity of sickle cell disease (SCD), but hematopoietic cell transplantation (HCT), and more recently, autologous gene therapy are the only treatments that have curative potential for SCD. While registry-based studies provide population-level estimates, they do not address the uncertainty regarding individual outcomes of HCT. Computational machine learning (ML) has the potential to identify generalizable predictive patterns and quantify uncertainty in estimates, thereby improving clinical decision-making. There is no existing ML model for SCD, and ML models for HCT for other diseases focus on single outcomes rather than all relevant outcomes.

**Objective:**

This study aims to address the existing knowledge gap by developing and validating an individualized ML prediction model SPRIGHT (Sickle Cell Predicting Outcomes of Hematopoietic Cell Transplantation), incorporating multiple relevant pre-HCT features to make predictions of key post-HCT clinical outcomes.

**Methods:**

We applied a supervised random forest ML model to clinical parameters in a deidentified Center for International Blood and Marrow Transplant Research (CIBMTR) dataset of 1641 patients who underwent HCT between 1991 and 2021 and were followed for a median of 42.5 (IQR 52.5;range 0.3‐312.9) months. We applied forward and reverse feature selection methods to optimize a set of predictive variables. To counter the imbalance bias toward predicting positive outcomes due to the small number of negative outcomes, we constructed a training dataset, taking each outcome as variable of interest, and performed 2-times repeated 10-fold cross-validation. SPRIGHT is a web-based individualized prediction tool accessible by smartphone, tablet, or personal computer. It incorporates predictive variables of age, age group, Karnofsky or Lansky score, comorbidity index, recipient cytomegalovirus seropositivity, history of acute chest syndrome, need for exchange transfusion, occurrence and frequency of vaso-occlusive crisis (VOC) before HCT, and either a published or custom chemotherapy or radiation conditioning, serotherapy, and graft-versus-host disease prophylaxis. SPRIGHT makes individualized predictions of overall survival (OS), event-free survival, graft failure, acute graft-versus-host disease (AGVHD), chronic graft-versus-host disease (CGVHD), and occurrence of VOC or stroke post-HCT.

**Results:**

The model's ability to distinguish between positive and negative classes, that is, discrimination, was evaluated using the area under the curve, accuracy, and balanced accuracy. Discrimination met or exceeded published predictive benchmarks with area under the curve for OS (0.7925), event-free survival (0.7900), graft failure (0.8024), acute graft-versus-host disease (0.6793), chronic graft-versus-host disease (0.7320), and VOC post-HCT (0.8779). SPRIGHT revealed good calibration with a slope of 0.87‐0.96, with small negative intercepts (–0.01 to 0.03), for 4 out of the 5 outcomes. However, OS exhibits nonideal calibration, which may be reflective of the overall high OS in all subgroups.

**Conclusions:**

A web-based ML prediction tool incorporating multiple clinically relevant variables predicts key clinical outcomes with a high level of discrimination and calibration and has potential in shared decision-making

## Introduction

The complications of sickle cell disease (SCD) can be prevented or ameliorated by disease-modifying therapies [1], but hematopoietic cell transplantation (HCT), and more recently, gene therapy remain the only therapeutic options with curative intent [2-12]. Population-level studies demonstrate the association of outcomes of HCT with age, type of donor, type of conditioning, and graft-versus-host disease (GVHD) prophylaxis [5,6], but do not address the uncertainty regarding individualized outcomes of HCT. Such uncertainty contributes to the decisional dilemma and is a barrier to shared decision-making. An individualized prediction model that incorporates all predictive variables and provides individualized estimates of key outcomes of HCT of interest to patients and their physicians has the potential to inform shared decision-making [13-15]. Brazauskas et al [16] have proposed a predictive model based on the age of the recipient and the type of donor. However, their model does not incorporate other clinically relevant patient, HCT, and disease characteristics and does not include all key outcomes. Computational machine learning (ML) has the potential to determine generalizable predictive patterns and quantify uncertainty, but published ML predictive models for HCT are limited to predicting single clinical outcomes [17-30]. To address the knowledge gap, we developed and described the initial validation of SPRIGHT (Sickle Cell Predicting Outcomes of Hematopoietic Cell Transplantation), an individualized ML prediction model for outcomes of HCT for SCD, incorporating multiple relevant features to make predictions of key clinical outcomes.

## Methods

### Dataset

We developed SPRIGHT using an anonymized HCT for the SCD dataset [31] derived from data submitted to the Center for International Bone Marrow Transplant Research (CIBMTR) registry on children and adults undergoing HCT for SCD between 1991 and 2021 in the United States. The dataset was obtained through the NHLBI (National Heart Lung and Blood Institute) Biologic Specimen and Data Repository Information Coordinating Center (BIOLINCC) [32].

The CIBMTR maintains a research database to serve as a comprehensive data source that can be used to study cellular therapies, including HCT. All US transplant centers are required to submit outcomes data on all allogeneic transplants when either the stem cell donation or the transplant occurs within the United States. CIBMTR assigns patients to either a Transplant Essential Data (TED) track, which collects core transplant data, or a Comprehensive Report Form (CRF) track that captures detailed disease- and treatment-related data [31]. Assignment to each track is based on submission of the initial pretransplantation TED 2000032419 form and uses a weighted randomization algorithm designed to produce a cohort representative of current clinical practice. All centers submit a Pre-HCT TED Form (Form 2400) for each allogeneic (related or unrelated) HCT.

Of 1641 patients undergoing HCT for SCD between 1991 and 2021, on whom data were submitted to CIBMTR, detailed CRFs were submitted on 763 patients. Of the patients in the dataset, 84% (1377/1641) had undergone HCT after 2007. We performed the imputation of missing data using MissForest, an ML data imputation algorithm that operates on random forest (RF).

### Feature Selection

We identified overall survival (OS), event-free survival (EFS), graft failure (GF), acute graft-versus-host disease (AGVHD), and chronic graft-versus-host disease (CGVHD) as key outcomes. We used wrapper methods of backward feature elimination (BFE) and forward sequential selection (FSS) to select and optimize the input variables [33,34]. The BFE procedure begins with a complete set of features and a chosen ML model. The model is trained, and the importance of each feature is evaluated based on the model’s coefficients or feature importance scores, and then the least important feature is discarded, and the model is retrained on the remaining features. This process is repeated until a predetermined number of features is reached or until further removal of features leads to a significant decrease in model performance. Using FSS, we incrementally built a feature set starting with an empty model, sequentially adding a feature that most improves the model performance at each step, as evaluated through a predefined metric like cross-validation score. We continued this stepwise addition until new features no longer significantly enhanced the model or a specified number of features was reached. We have included the detailed descriptive statistics and missingness for each of the selected features and outcomes of interest in Table S3 in Multimedia Appendix 1 and Table S4 in Multimedia Appendix 2

### Model Design

The model was designed by subsampling the majority class to create subtraining datasets, followed by pooling and thresholding to obtain the final prediction [35]. We determined the discriminative performance of the model, which refers to how well the predictions can separate between 2 groups of participants, that is, those with or without an outcome. Discrimination was quantified by the concordance (c) statistic (index), which for binary outcomes, is equivalent to the area under the curve (AUC). We also assessed accuracy, that is, the percentage of correct predictions out of all predictions correct or incorrect in the model. We also assessed balanced accuracy, which is an accuracy adjusted for imbalance and is derived by averaging sensitivity and specificity, so that each class’s importance is equal. We compared RF, extreme gradient boosting, logistic regression, Naive Bayes, AdaBoost, and support vector classification algorithms (Table 1). The HCT for SCD dataset spans children and adults undergoing HCT between 1991 and 2021. During this time, there have been many changes in conditioning regimens and improvements in supportive care. Gluckman et al [6] reported that EFS was higher in patients who underwent HCT in or after 2007 as compared to those who underwent HCT in or before 2006 (HR [hazard ratio] 0.95, CI 0.90‐0.99; *P*=.01). To determine if the model performed consistently across eras, we tested model performance in patients with the year of HCT <2007 versus HCT >2007. We also tested model performance across age at HCT <10, <18, and >18 years, respectively, including outcomes at 1- and 3-year post-HCT (Table 2).

**Table 1.** Model performance: comparison of area under the curve of different algorithms for each outcome.

Model	Outcome of interest				
	EFS^a^	OS^b^	GF^c^	AGVHD^d^	CGVHD^e^
Random forest entire dataset	0.7900	0.7925	0.8024	0.6793	0.7320
XGBoost^f^	0.7754	0.7785	0.7948	0.6731	0.7230
Logistic regression	0.7464	0.7835	0.7578	0.6925	0.7019
Naïve Bayes	0.6930	0.7111	0.7107	0.6386	0.6384
Adaboost	0.7452	0.7806	0.7561	0.6934	0.7005
Support vector classifier	0.7357	0.7810	0.7561	0.6841	0.7061

a EFS: event-free survival.

b OS: overall survival.

c GF: graft failure.

d AGVHD: acute graft-versus-host disease.

e CGVHD: chronic graft-versus-host disease.

f XGBoost: extreme gradient boosting.

**Table 2.** Model performance: Comparison of area under the curve across different paradigms and data time periods.

Paradigm	Outcome of interest				
	EFS^a^	OS^b^	GF^c^	AGVHD^d^	CGVHD^e^
All data	0.790	0.793	0.802	0.679	0.732
Post 2007 data	0.787	0.775	0.783	0.702	0.729
1 Year outcome analysis on post 2007 data	0.801	0.788	0.807	0.741	0.705
3 Year outcome analysis on post 2007	0.792	0.771	0.82	0.730	0.721

a EFS: event-free survival.

b OS: overall survival.

c GF: graft failure.

d AGVHD: acute graft-versus-host disease.

e CGVHD: chronic graft-versus-host disease.

The accuracy of risk estimates, relating to the agreement between the estimated and observed number of events, is called “calibration [36,37].” Calibration is crucial in predictive algorithms because it ensures the accuracy of risk estimates, which directly affects clinical decision-making and patient expectations. Poor calibration can lead to systematic overestimation or underestimation of risk, resulting in false expectations and potentially harmful decisions [36]. We performed causal isotonic calibration, a novel nonparametric method for calibrating predictors of heterogeneous treatment effect [37]. We performed a 5-fold internal cross-validation on the training set to determine the optimal calibration. The resultant calibration model was then applied to the predictions during evaluation. To adjust for the bias caused due to undersampling, we recalibrate the probabilities according to the method by Pozollo et al [38]. We evaluated the calibration curve, slope, and intercept across different outcomes of interest in the post-2007 data (Table 3).

**Table 3.** Calibration analysis: slope and intercept across different outcomes of interest for post-2007 data.

Outcome of interest	Calibration property	
	Slope	Intercept
EFS^a^	0.93	–0.02
OS^b^	0.75	–0.07
GF^c^	0.9	–0.02
AGVHD^d^	0.96	–0.03
CGVHD^e^	0.87	–0.01

a EFS: event-free survival.

b OS: overall survival.

c GF: graft failure.

d AGVHD: acute graft-versus-host disease.

e CGVHD: chronic graft-versus-host disease.

To understand the contributions of each feature to the predictive model, we use the Shapley additive explanations (SHAP) scores (MultimediaAppendices 3^7^). SHAP scores are based on game theory’s Shapley values, quantifying each feature’s marginal contribution to individual predictions. They are calculated by measuring how each feature affects the model output when included or excluded from all possible feature combinations.

### The Problem of Imbalance

**Figure 1.**
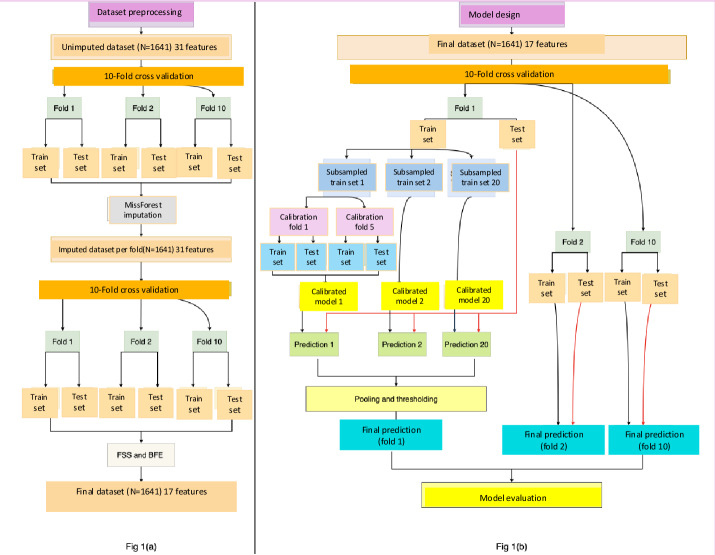
Data partitioning, imputation, cross-validation, and item optimization A) Dataset preprocessing, imputation, and feature selection. (B) Model finalization and test of predictive performance. BFE: backward feature elimination; FSS: forward sequential selection.

The outcomes data for HCT for SCD is imbalanced, with very few negative outcomes. This imbalance has the potential to lead to a prediction bias, where an uncorrected model default may be skewed toward predicting positive outcomes. To address the problem of imbalance, we constructed a training dataset taking each outcome as variable of interest. We included randomly sampled positive outcomes, typically 1.5‐3 times the total instances of the variable of interest. To address the issue of class imbalance, we used a 2-step approach involving bootstrapping and consensus-based decision-making. Initially, we generated 20 bootstrapped datasets by undersampling the majority class to achieve a (2-3):1 ratio with the minority class. These datasets served as the training sets for our predictive models, ensuring a balanced representation of classes during model training. Once the models were trained, each was then tested on a consistent test dataset to obtain a series of predictions. These individual predictions were subsequently pooled across all models. A final prediction model for each test instance was determined based on a consensus threshold. If the majority of the models exceeded a predetermined threshold agreed on a particular class, that class was assigned as the outcome for the instance. We ran the test dataset and used a RF algorithm on a 2-times repeated 10-fold cross-validation to demonstrate our model’s versatility and response to unknown data (Figure 1). We assigned value 1 for a negative outcome prediction and −1 for a positive outcome prediction and found the average sum across the 20 trials for each element.

Throughout the paper, we are guided by the CREMLS (Consolidated Reporting of Machine Learning Studies) guidelines [39], to ensure transparency and rigor in reporting. We have attached a completed author CREMLS checklist.

### Ethical Considerations

The Institutional Review Board at Yale University determined on March 3, 2033, that this study did not constitute human participants research per IRB protocol number 2000032419.

## Results

### Feature Selection and Optimization

Of the 160 variables in the dataset, we selected 31 potentially clinically relevant predictive variables. Through FSS and BFE processes, we selected a final set of 17 predictive variables grouped into 3 categories. Patient Data variables included age at transplant, age group at transplant, sex, Karnofsky or Lansky score, HCT-comorbidity index, and recipient CMV serostatus. SCD variables included Number of ACS syndromes within 2 years pre-HCT, Required exchange transfusion, Vaso-occlusive crisis needing hospitalization in 2 years pre-HCT, and Hospitalization frequency for vaso-occlusive crises. Transplant Data variables included Donor type, Graft type, Conditioning intensity, Conditioning regimen, Serotherapy (ATG or Alemtuzumab), GVHD prophylaxis, and Donor-recipient HLA matching.

### Evaluation Outcomes and Model Performance

Discrimination in predictive performance is evaluated using accuracy, the percentage of correct predictions out of all predictions, balanced accuracy, the average sensitivity and specificity, and each class’s importance is equal, and AUC, the measure of a model’s true positive rate against a false positive rate, indicates the ability to differentiate classes. AUC is the metric used in published literature. The RF model achieved the highest predictive AUC (Table 1) across multiple clinical outcomes. We measure our model’s performance using the benchmark established in the literature. To determine the statistical validity, we implemented the method proposed by Bouckaert and Frank [40]. We first performed 2×10 repeated cross-validation and obtained the performance of each of the models for each fold. We then applied Nadeau and Bengio’s [41] correction, which accounts for training set overlap in the variance estimation, to check whether the mean AUC of the RF is greater than the mean AUC of the other models across folds. The differences in the mean AUC were statistically significant for EFS, OS, GF, AGVHD, and CGVHD (*P*<.05). We performed hyperparameter tuning using grid search cross-validation for the RF model. The ideal hyperparameters are described in Table 4. AUC, accuracy, and balanced accuracy equaled or exceeded the benchmarks of the ML predictive tools in the published literature [16,42]. The RF model has been previously reported to have the best AUC in predicting survival following HCT [24]. Brazauskas et al [16] published benchmark AUC for EFS of 0.72, and Taheriyan et al [42] reported benchmarks AUC 0.82 for AGVHD post-HCT. Accuracy and balanced accuracy were excellent for EFS (0.76, 0.69), OS (0.82, 0.68), GF (0.8, 0.71), vaso-occlusive pain post-HCT (0.9, 0.78), stroke post-HCT (0.92, 0.65), acute GVHD (0.71, 0.60), CGVHD (0.72, 0.63).

**Table 4.** Final tuned hyperparameters of the random forest model for each outcome.

Outcome of interest	Random forest hyperparameters					
	Max depth	Min samples split	Min samples leaf	Criterion	CCP_alpha^a^	Max features
EFS^b^	20	10	16	entropy	0.0	0.7
OS^c^	20	10	8	entropy	0.01	0.125
GF^d^	20	20	16	entropy	0.0	0.5
AGVHD^e^	5	20	8	entropy	0.0	0.5
CGVHD^f^	15	10	8	entropy	0.0	0.5

a CCP_alpha: cost complexity pruning alpha.

b EFS: event-free survival.

c OS: overall survival.

d GF: graft failure.

e AGVHD: acute graft-versus-host disease.

f CGVHD: chronic graft-versus-host disease.

SPRIGHT retained high AUC in subpopulations, including patients ≤10, ≤18, >18 of age in undergoing HCT after 2007, as well as 1- and 3-year survival analysis (Table 1).

**Figure 2.**
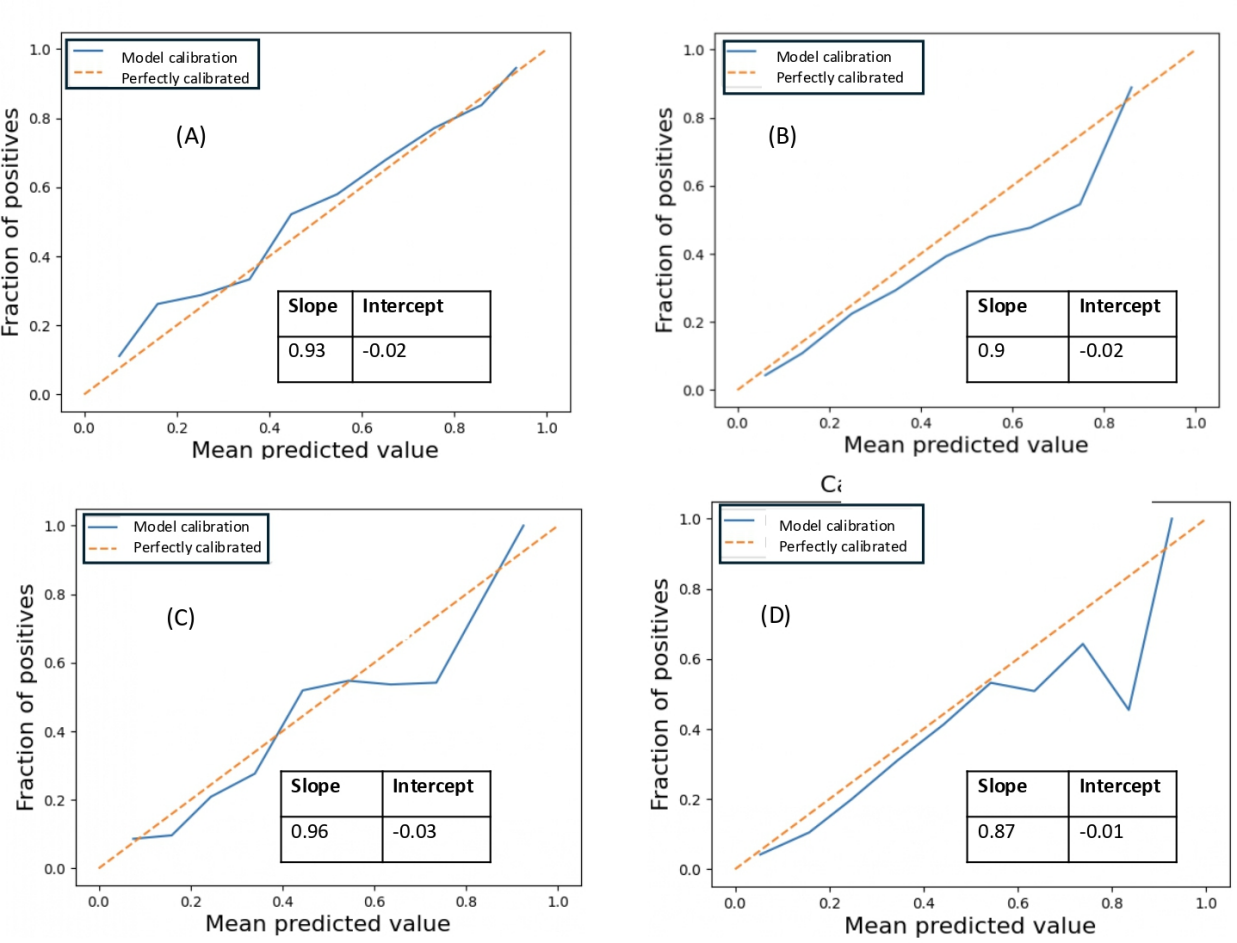
Calibration of SPRIGHT for various outcomes. Calibration curve, slope, and intercept for (A). Event-free survival, (B) graft failure, (C) acute graft-versus-host disease (GVHD), and (D) chronic GVHD. Overall survival (OS) shows a nonideal calibration slope of 0.75 and intercept of −0.07 (data not shown) and may be reflective of the overall high OS in all subgroups.

Calibration is the agreement between the estimated and observed number of events, for major outcomes. A calibration slope of 1 and an intercept close to zero are associated with good calibration. SPRIGHT revealed good calibration with a slope range of 0.87‐0.96, with small negative intercepts (−0.01 to 0.03), for 4 out of the 5 outcomes. However, OS exhibits nonideal calibration and may be reflective of the overall high OS in all subgroups (Figure 2).

Feature importance analysis using SHAP values revealed consistent patterns across all outcomes. Age at transplantation and donor type emerged as the most influential predictors, corroborating previous findings by Brazauskas et al [16] and Eapen et al [5]. Disease severity indicators, most importantly the frequency of acute chest syndrome episodes in the 2 years preceding HCT, were identified as another critical predictor. The frequency of VOC requiring hospitalization and the need for exchange transfusions also demonstrated substantial predictive importance. This suggests the utility of including pretransplant disease characteristics for predicting outcomes. The comprehensive SHAP analysis, including feature importance rankings and their relative contributions to model predictions, is presented in MultimediaAppendices 3^7^.

To demonstrate the clinical utility of our model, we analyzed predictions across 3 distinct hypothetical patient scenarios. Case-specific patient characteristics and their corresponding predicted outcomes are detailed in Table 5 Table S3 in Multimedia Appendix 8 respectively. The model’s predictions aligned with established clinical observations, showing less favorable outcomes in cases involving non-HLA identical donors and in older patients with more severe disease characteristics, which were consistent with previous studies [5,16].

**Table 5.** Hypothetical patient profiles with varying age, donor type, and disease severity.

Data	Patient 1	Patient 2	Patient 3
Patient data			
Age at transplant (year)	6	6	16
Age group (year)	≤10	≤10	11-17
Sex	Male	Male	Male
KPS or Lansky^a^ score	<90	<90	<90
HCT^b^-Comorbidity index (in range)	0-2	0-2	0-2
Recipient CMV^c^ serostatus	Negative	Negative	Negative
Transplant data			
Donor	HLA^d^ identical sibling	HLA mismatch relative	HLA identical sibling
Graft type	Bone marrow	Bone marrow	Bone marrow
Donor-recipient HLA matching	8/8	7/8	8/8
Conditioning intensity	Myeloablative	Nonmyeloablative	Myeloablative
Conditioning regimen	Flu/Bu^e^	TBI/Cy/Flu/TT^f^	Flu/Bu
Serotherapy	ATG^g^	ATG	ATG
GVHD^h^ prophylaxis	CNI+MTX^i^	Post-Cy+Siro± MMF^j^	CNI+MTX
SCD^k^ data			
Number of ACS^l^ syndromes within 2 year pre-HCT	0	0	2
Require exchange transfusion	No	No	No
VOC^m^ requiring hospitalization within 2 year pre-HCT	No	No	Yes
Frequency of hospitalizations for VOC	<3 per year	<3 per year	<3 per year

a KPS: Karnofsky performance status.

b HCT: hematopoietic cell transplantation.

c CMV: cytomegalovirus.

d HLA: human leukocyte antigen.

e Flu/Bu: fludarabine + busulfan.

f TBI/Cy/Flu/TT: total body irradiation + cyclophosphamide + fludarabine + thiotepa.

g ATG: anti-thymocyte globulin.

h GVHD: graft-versus-host disease.

i CNI+MTX: calcineurin inhibitor + methotrexate.

j Post-Cy+Siro± MMF: post-transplant cyclophosphamide + sirolimus + mycophenolate mofetil.

k SCD: sickle cell disease.

l ACS: acute chest syndrome.

m VOC: vaso-occlusive crisis.

### SPRIGHT User Interface

**Figure 3.**
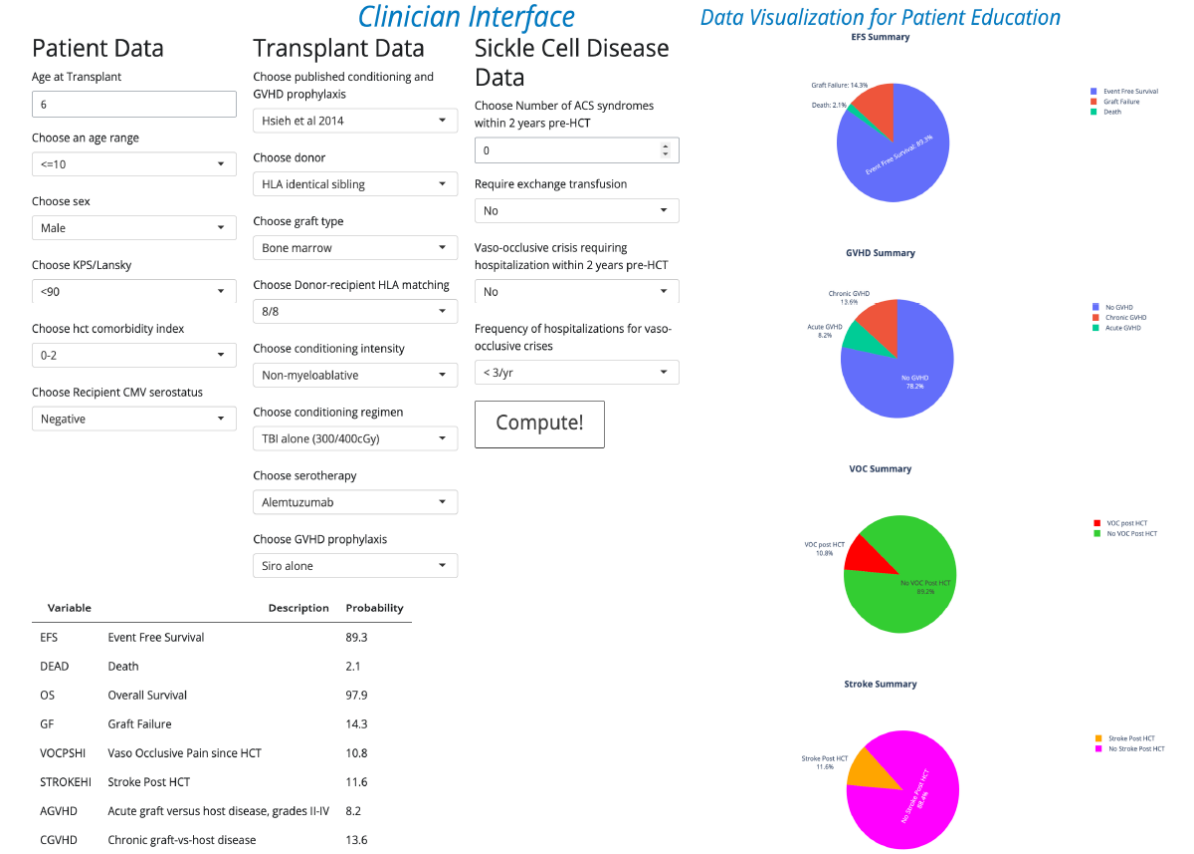
Clinician user interface of sickle cell predicting outcomes of hematopoietic cell transplantation

Age is the only numeric feature that is entered manually. The rest of the inputs are categorical and are entered by selecting an option from a drop-down menu. Patient-specific, disease-specific data, and treatment donor, one of the major published treatment regimens or a customized conditioning regimen, conditioning intensity, and GVHD prophylaxis ATG/Alemtuzumab data can be entered. The tabular output describes predicted OS, EFS, GF, Death, AGVHD, CGVHD, VOC, and stroke. The predicted outcomes are also pictorially represented in pie charts. One unique feature is that the user has the option of selecting a published HCT regimen or selecting a custom regimen by combining conditioning, GVHD prophylaxis, and serotherapy (Figure 3; Table S4 in Multimedia Appendix 9). This feature decreases keystrokes, improves ease of use of the app, and facilitates a comparative analysis across donor types and treatment regimens for different donor types. SPRIGHT can be accessed on any smartphone, tablet, or personal computer using a shortened URL or a QR code. The individualized outcomes are also represented as pie charts displaying individualized estimates (Figure 3). The pie charts can be downloaded and shared with the patient or added to the electronic medical record as an image.

## Discussion

### Principal Findings

We describe the development and internal validation of SPRIGHT, which, to the best of our knowledge, is the first ML individualized prediction tool for HCT for SCD. Eapen et al [5] identified age, donor type, and conditioning regimen intensity as critical predictive factors of outcomes of HCT for SCD. Gluckman et al [6] identified age and year of transplant as critical factors. Younger patients were shown to have higher EFS. Cappelli et al [43] reported better OS and EFS and a lower incidence of AGVHD and CGVHD in younger patients. Together, these registry-based studies generated important population-level predictive factors of HCT for SCD. They do not, however, provide a means to combine patient, transplant, and disease characteristics into a personalized predictive model for outcomes of HCT. The SPRIGHT prediction model incorporates multiple relevant pre-HCT predictive factors for the individualized production of key clinically relevant post-HCT outcomes. The RF algorithm outperforms the Brazauskas model, other ML algorithms, and logistic regression in predictive performance. The RF-based SPRIGHT prediction model has high predictive discrimination and calibration performance. The excellent discriminative predictive performance is demonstrated by the high value of AUC across all outcomes across all eras, age groups, and follow-up periods of 1 or 3 years. Going beyond the commonly reported predictive discrimination with AUC, we also reported accuracy and balanced accuracy and calibration measures of calibration curve, slope, and intercept. An important innovation of SPRIGHT is the option for the end user to select a published regimen that combines chemotherapy or radiation conditioning, serotherapy, and GVHD prophylaxis. This innovation simplifies the decision-making process for clinicians and allows them to compare potential outcomes across different regimens and donor types. Thus, SPRIGHT helps physicians and patients in discerning the nuances in efficacy and safety of HCT for the individual and has the potential to inform and guide shared decision-making. To mitigate overfitting and validate model performance, we used 10-fold cross-validation, ensuring robustness by mimicking multiple tests on independent datasets. This method approximates the effectiveness of external validation by exposing the model to various training and validation splits, thus predicting its behavior on unseen data. We addressed potential feature collinearity using recursive feature elimination for feature selection and RFs, which inherently mitigate correlation effects through random feature subsampling at each tree.

### Limitations

There are several limitations to this study. The SPRIGHT predictive tool is based on the HCT for the SCD dataset derived from data reported to CIBMTR. The use of the CIBMTR dataset allows us to access the entire US experience reported, but it is also subject to limitations of registry studies, including bias, loss to follow-up, and a lack of generalizability across era, center volume, and expertise. While SPRIGHT uses multiple predictive variables, it is limited to those variables collected by CIBMTR. In 2019, Bolanos-Meade et al [44] reported an improved EFS following mismatched relative donor HCT as compared to their previous report in 2009 following the escalation of the dose of total body irradiation from 200 to 400 cGy [6,45]. However, CIBMTR form 2400 and consequently the HCT for the SCD dataset do not distinguish between patients who received the 2 dose levels of total body irradiation. Further, since pain crisis post-HCT is included as a discrete variable in the HCT for the SCD dataset, it is not possible to discern its timing, frequency, or severity. In the CIBMTR dataset, an analysis of predictor completeness reveals that 10/13 TED variables chosen have a completion rate exceeding 98%. However, only approximately 46.5% (763/1641) of patients are on the CRF track, with comprehensive disease-specific data available, a category under which 4 of our predictor variables fall. While the missing data could be a source of bias, the missingness of data was only a function of whether the institution was designated as a TED-only or CRF and whether the CIBMTR algorithm assigned an individual patient to the CRF track. Thus, the missing data may be missing at random. The model we used for imputation, MissForest, has been shown to outperform all other algorithms in all metrics [46,47]. However, these imputation algorithms can produce severely biased regression coefficients and require a careful critique of the missing data mechanism and the interrelationships between the variables in the data [48]. Overall, we acknowledge the concerns regarding the lack of details of SCD-related complications and the completeness of reporting of SCD-related clinical outcomes in the CIBMTR dataset. We, however, also recognize that the CIBMTR registry, with federally mandated data submission, contains the most complete data available. We support ongoing efforts to refine the data collection measures and training of data collection staff. One of the limitations of our model is the rarity of death events in the dataset, which limits the precision of calibration for OS. As a result, the model tends to slightly overestimate OS risk, particularly in subgroups with fewer events. This calibration limitation should be considered when interpreting OS predictions. Across all outcomes, despite mitigatory efforts to combat the inherent bias due to class imbalance, the bias may not be fully eradicated.

The tool incorporates a set of treatment regimens that have been carefully selected from peer-reviewed studies, ensuring that they are backed by sufficient clinical data. These regimens provide reliable and evidence-based predictions. However, custom combinations that are entered by users may fall outside of the dataset’s training and may not be fully supported by the underlying data. In such cases, it is essential to rely on clinical expertise and user discretion when interpreting the predictions.

We recognize a limitation in the registry data due to inherent selection bias, with limited insight into the clinical reasoning behind regimen choices. While the data detail the regimens administered, the underlying clinical judgment is often not recorded. This limitation is particularly notable in HCT, where the absence of universally established standards of care presents additional challenges. Thus, the model and its results should be interpreted within this context.

### Comparison With Prior Work

Brazauskas et al [16] developed a risk score using age and donor type as discrete variables. They reported that patients aged ≤12 years with an HLA-matched sibling donor were at the lowest risk. Patients aged ≥13 years with an HLA-matched sibling donor or aged ≤12 years with an HLA-matched unrelated donor were at intermediate risk. All other groups were at high risk. This simple risk score has good predictive performance but has certain gaps that limit its utility in the individualized prediction of outcomes. Gluckman et al [6] used age as a continuous variable and observed that for every 1-year increment in age, there was a 9% increase in the HR for treatment failure (graft failure or death) and a 10% increase in the HR for death. Thus, the Brazauskas model does not include the potential predictive value of increasing age from 5 to 13 years. Further, Brazauskas et al [16] do not incorporate other patient, disease, and conditioning regimen characteristics in the prediction model because they considered these factors to be dynamic and subject to change. The Brazauskas model limits the predicted post-HCT outcomes to death, OS, and EFS and does not include other outcomes that are important to physicians and patients in shared decision-making, such as AGVHD, CGVHD, recurrent pain crisis, or stroke after HCT [13,49-51]. SPRIGHT predicts these outcomes with high predictive performance. Other published ML predictive models for HCT for other diseases are of limited clinical relevance in decision-making since they limit themselves to predicting single outcomes, such as death, overall survival, disease relapse, GVHD, busulfan exposure, kidney injury, or reactivation of Epstein-Barr virus [17-30].

In developing SPRIGHT, we addressed the gaps in the knowledge in individualized prediction of outcomes of HCT. We included 7 clinically relevant outcomes, including rates of OS, EFS, GF, death, AGVHD, CGVHD, and VOC. Each of these 7 clinical outcomes initially required a distinct set of 10‐11 pre-HCT predictive features for optimal performance, leading to incomplete overlap and potential model fragmentation. For addressing this, we adopted a unified approach by selecting a comprehensive set of 17 pre-HCT patient, HCT, and disease characteristics. By applying robust feature selection techniques to optimize predictive performance and improve the model’s overall clinical applicability, we demonstrated the predictive value of these features. Further, patient and disease features inform patient selection and HCT features inform regimen selection. Thus, these additional pre-HCT features are important, clinically relevant considerations in decision-making.

### Future Directions

We present here an initial in-lab validation of the predictive model. We recognize that the use of a US-based dataset may limit the generalizability of our findings to other regions or health care systems due to potential differences in population characteristics and treatment protocols. For further external validation across different geographic locations to establish the model’s generalizability and clinical utility, we propose to use the European Bone Marrow Transplantation Registry (EBMT) dataset, a completely independent dataset, in collaboration with European investigators. Of note, Gluckman et al [6] have previously combined CIBMTR and EBMT registry data on SCD for analysis and included similar numbers of children, adults, and donor types from the 2 registries and do not report differences in predictive factors or outcomes in the 2 registries. We have demonstrated that the predictive performance remains equivalent whether we use the entire dataset or the more recent data after 2007, which represents 84% (1378/1641) of participants in the dataset. Acknowledging the evolving nature of supportive care practices in HCT, we propose further temporal validation with future years of data being added to this dataset. We also propose to continue to enhance SPRIGHT by incorporating expert opinion, adapting to patient health literacy, values, and preferences, and using patient-friendly data visualization to support shared decision-making [52-54].

### Conclusions

In conclusion, the SPRIGHT prediction model integrates individual-specific patient and disease characteristics, conditioning regimens, GVHD prophylaxis, and donor characteristics and predicts key clinical outcomes. It exhibits superior predictive performance across multiple measures of discrimination and calibration as compared to logistic regression and other ensemble ML methods.

## Supplementary material

10.2196/64519 Multimedia Appendix 1 Feature distribution of the dataset.

10.2196/64519 Multimedia Appendix 2 Outcome distribution.

10.2196/64519 Multimedia Appendix 3 Mean absolute Shapley additive explanations values quantifying predictor importance for acute graft-vs-host disease. Values averaged across cross-validation folds and bootstraps.

10.2196/64519 Multimedia Appendix 4 Mean absolute Shapley additive explanations values quantifying predictor importance for graft failure. Values averaged across cross-validation folds and bootstraps.

10.2196/64519 Multimedia Appendix 5 Mean absolute Shapley additive explanations values quantifying predictor importance for Event Free Survival. Values averaged across cross-validation folds and bootstraps.

10.2196/64519 Multimedia Appendix 6 Mean absolute Shapley additive explanations values quantifying predictor importance for CGVHD. Values averaged across cross-validation folds and bootstraps.

10.2196/64519 Multimedia Appendix 7 Mean absolute Shapley additive explanations values quantifying predictor importance for overall survival. Values averaged across cross-validation folds and bootstraps.

10.2196/64519 Multimedia Appendix 8 Predicted probability percentage outcomes for each of the hypothetical patient profiles.

10.2196/64519 Multimedia Appendix 9 Combinations of conditioning regimen, serotherapy, and graft-versus-host disease prophylaxis used in published case series, which are included in the SPRIGHT.

10.2196/64519 Checklist 1 Consolidated Reporting of Machine Learning Studies checklist.

## Abbreviations

AGVHD: acute graft-versus-host disease
AUC: area under the curve
BFE: backward feature elimination
BIOLINCC: Biologic Specimen and Data Repository Information Coordinating Center
CGVHD: chronic graft-versus-host disease
CIBMTR: Center for International Bone Marrow Transplant Research
CREMLS: Consolidated Reporting of Machine Learning Studies
CRF: Comprehensive Report Form
EBMT: European Bone Marrow Transplantation Registry
EFS: event-free survival
FSS: forward sequential selection
GF: graft-free survival
GVHD: graft-versus-host disease
HCT: hematopoietic cell transplantation
HR: hazard ratio
ML: machine learning
NHLBI: National Heart, Lung, and Blood Institute
OS: overall survival
SCD: sickle cell disease
SHAP: Shapley additive explanations
SPRIGHT: Sickle Cell Predicting Outcomes of Hematopoietic Cell Transplantation
VOC: vasocclusive crisis

## References

[1] Kato GJ, Piel FB, Reid CD (2018). Sickle cell disease. Nat Rev Dis Primers.

[2] Walters MC, De Castro LM, Sullivan KM (2016). Indications and results of HLA-identical sibling hematopoietic cell transplantation for sickle cell disease. Biol Blood Marrow Transplant.

[3] Walters MC, Patience M, Leisenring W (1996). Bone marrow transplantation for sickle cell disease. N Engl J Med.

[4] Walters MC, Patience M, Leisenring W (1997). Collaborative multicenter investigation of marrow transplantation for sickle cell disease: current results and future directions. Biol Blood Marrow Transplant.

[5] Eapen M, Brazauskas R, Walters MC (2019). Effect of donor type and conditioning regimen intensity on allogeneic transplantation outcomes in patients with sickle cell disease: a retrospective multicentre, cohort study. Lancet Haematol.

[6] Gluckman E, Cappelli B, Bernaudin F (2017). Sickle cell disease: an international survey of results of HLA-identical sibling hematopoietic stem cell transplantation. Blood.

[7] Hsieh MM, Fitzhugh CD, Weitzel RP (2014). Nonmyeloablative HLA-matched sibling allogeneic hematopoietic stem cell transplantation for severe sickle cell phenotype. JAMA.

[8] Krishnamurti L, Neuberg DS, Sullivan KM (2019). Bone marrow transplantation for adolescents and young adults with sickle cell disease: results of a prospective multicenter pilot study. Am J Hematol.

[9] Shenoy S, Eapen M, Panepinto JA (2016). A trial of unrelated donor marrow transplantation for children with severe sickle cell disease. Blood.

[10] King AA, Kamani N, Bunin N (2015). Successful matched sibling donor marrow transplantation following reduced intensity conditioning in children with hemoglobinopathies. Am J Hematol.

[11] Gluckman E, Cappelli B, Scigliuolo GM, Fuente JD la, Corbacioglu S (2020). Alternative donor hematopoietic stem cell transplantation for sickle cell disease in Europe. Hematol Oncol Stem Cell Ther.

[12] Patel DA, Akinsete AM, Fuente J de la, Kassim AA (2020). Haploidentical bone marrow transplant with posttransplant cyclophosphamide for sickle cell disease: an update. Hematol Oncol Stem Cell Ther.

[13] Sinha CB, Bakshi N, Ross D, Loewenstein G, Krishnamurti L (2021). Primary caregiver decision-making in hematopoietic cell transplantation and gene therapy for sickle cell disease. Pediatr Blood Cancer.

[14] Bakshi N, Katoch D, Sinha CB (2020). Assessment of patient and caregiver attitudes and approaches to decision-making regarding bone marrow transplant for sickle cell disease: a qualitative study. JAMA Netw Open.

[15] Veludhandi A, Ross D, Sinha CB, McCracken C, Bakshi N, Krishnamurti L (2021). A decision support tool for allogeneic hematopoietic stem cell transplantation for children with sickle cell disease: acceptability and usability study. JMIR Form Res.

[16] Brazauskas R, Scigliuolo GM, Wang HL (2020). Risk score to predict event-free survival after hematopoietic cell transplant for sickle cell disease. Blood.

[17] Afanaseva KS, Bakin EA, Smirnova AG (2023). A pilot study of implication of machine learning for relapse prediction after allogeneic stem cell transplantation in adults with Ph-positive acute lymphoblastic leukemia. Sci Rep.

[18] Al-Riyami AZ, Maryamchik E, Hanna RS (2023). A machine-learning model that incorporates CD45 surface expression predicts hematopoietic progenitor cell recovery after freeze-thaw. Cytotherapy.

[19] Fan S, Hong HY, Dong XY (2023). Machine learning algorithm as a prognostic tool for Epstein-Barr virus reactivation after haploidentical hematopoietic stem cell transplantation. Blood Sci.

[20] Keret S, Rimar D, Lansiaux P (2023). Differentially expressed genes in systemic sclerosis: towards predictive medicine with new molecular tools for clinicians. Autoimmun Rev.

[21] Li D, Zhao J, Xu B (2023). Predicting busulfan exposure in patients undergoing hematopoietic stem cell transplantation using machine learning techniques. Expert Rev Clin Pharmacol.

[22] Mushtaq AH, Shafqat A, Salah HT, Hashmi SK, Muhsen IN (2023). Machine learning applications and challenges in graft-versus-host disease: a scoping review. Curr Opin Oncol.

[23] Shourabizadeh H, Aleman DM, Rousseau LM, Law AD, Viswabandya A, Michelis FV (2024). Machine learning for the prediction of survival post-allogeneic hematopoietic cell transplantation: a single-center experience. Acta Haematol.

[24] Sobrino S, Magnani A, Semeraro M (2023). Severe hematopoietic stem cell inflammation compromises chronic granulomatous disease gene therapy. Cell Rep Med.

[25] Sorror ML (2023). The use of prognostic models in allogeneic transplants: a perspective guide for clinicians and investigators. Blood.

[26] Sparapani RA, Logan BR, Maiers MJ, Laud PW, McCulloch RE (2023). Nonparametric failure time: Time-to-event machine learning with heteroskedastic Bayesian additive regression trees and low information omnibus Dirichlet process mixtures. Biometrics.

[27] von Asmuth EGJ, Neven B, Albert MH (2023). Predicting Patient Death after Allogeneic Stem Cell Transplantation for Inborn Errors Using Machine Learning (PREPAD): A European Society for Blood and Marrow Transplantation Inborn Errors Working Party Study. Transplant Cell Ther.

[28] Wang P, Liu C, Wei Z (2023). Nomogram for predicting early mortality after umbilical cord blood transplantation in children with inborn errors of immunity. J Clin Immunol.

[29] Zhou Y, Smith J, Keerthi D (2024). Longitudinal clinical data improve survival prediction after hematopoietic cell transplantation using machine learning. Blood Adv.

[30] Krishnamurti L, Liang J, He Z (2024). Incidence and risk factors of pain crisis after hematopoietic cell transplantation for sickle cell disease. Blood Adv.

[31] Friedman D, Dozor AJ, Milner J (2021). Stable to improved cardiac and pulmonary function in children with high-risk sickle cell disease following haploidentical stem cell transplantation. Bone Marrow Transplant.

[32] Giffen CA, Carroll LE, Adams JT, Brennan SP, Coady SA, Wagner EL (2015). Providing contemporary access to historical biospecimen collections: Development of the NHLBI Biologic Specimen and Data Repository Information Coordinating Center (BioLINCC). Biopreserv Biobank.

[33] Hong F, Tian L, Devanarayan V (2023). Improving the robustness of variable selection and predictive performance of regularized generalized linear models and Cox proportional hazard models. Mathematics.

[34] Jain R, Xu W (2023). Artificial Intelligence based wrapper for high dimensional feature selection. BMC Bioinformatics.

[35] Ruisen L, Songyi D, Chen W Bagging of XGBoost classifiers with random under-sampling and Tomek link for noisy label-imbalanced data. IOP Conf Ser: Mater Sci Eng.

[36] Van Calster B, McLernon DJ, van Smeden M, Wynants L, Steyerberg EW, Topic Group ‘Evaluating diagnostic tests and prediction models’ of the STRATOS initiative (2019). Calibration: the Achilles heel of predictive analytics. BMC Med.

[37] van der Laan L, Ulloa-Pérez E, Carone M, Luedtke A (2023). Causal isotonic calibration for heterogeneous treatment effects. Proc Mach Learn Res.

[38] Pozzolo AD, Caelen O, Johnson RA, Bontempi G Calibrating probability with undersampling for unbalanced classification.

[39] El Emam K, Leung TI, Malin B, Klement W, Eysenbach G (2024). Consolidated Reporting Guidelines for Prognostic and Diagnostic Machine Learning Models (CREMLS). J Med Internet Res.

[40] Bouckaert RR, Frank E (2004). Evaluating the Replicability of Significance Tests for Comparing Learning Algorithms.

[41] Nadeau C, Bengio Y (2003). Inference for the generalization error. Mach Learn.

[42] Taheriyan M, Safaee Nodehi S, Niakan Kalhori SR, Mohammadzadeh N (2022). A systematic review of the predicted outcomes related to hematopoietic stem cell transplantation: focus on applied machine learning methods’ performance. Expert Rev Hematol.

[43] Cappelli B, Volt F, Tozatto-Maio K (2019). Risk factors and outcomes according to age at transplantation with an HLA-identical sibling for sickle cell disease. Haematologica.

[44] Bolaños-Meade J, Cooke KR, Gamper CJ (2019). Effect of increased dose of total body irradiation on graft failure associated with HLA-haploidentical transplantation in patients with severe haemoglobinopathies: a prospective clinical trial. Lancet Haematol.

[45] Bolaños-Meade J, Brodsky RA (2014). Blood and marrow transplantation for sickle cell disease: is less more?. Blood Rev.

[46] Buczak P, Chen JJ, Pauly M (2023). Analyzing the effect of imputation on classification performance under MCAR and MAR mssing mechanisms. Entropy (Basel).

[47] Tang F, Ishwaran H (2017). Random forest missing data algorithms. Stat Anal Data Min.

[48] Hong S, Lynn HS (2020). Accuracy of random-forest-based imputation of missing data in the presence of non-normality, non-linearity, and interaction. BMC Med Res Methodol.

[49] Gluckman E, Fuente J de la, Cappelli B (2020). The role of HLA matching in unrelated donor hematopoietic stem cell transplantation for sickle cell disease in Europe. Bone Marrow Transplant.

[50] Meier ER, Dioguardi JV, Kamani N (2015). Current attitudes of parents and patients toward hematopoietic stem cell transplantation for sickle cell anemia. Pediatr Blood Cancer.

[51] Sinha CB, Meacham LR, Bakshi N, Ross D, Krishnamurti L (2023). Parental perspective on the risk of infertility and fertility preservation options for children and adolescents with sickle cell disease considering hematopoietic stem cell transplantation. Pediatr Blood Cancer.

[52] Greenes RA, Bates DW, Kawamoto K, Middleton B, Osheroff J, Shahar Y (2018). Clinical decision support models and frameworks: seeking to address research issues underlying implementation successes and failures. J Biomed Inform.

[53] Marcial LH, Richardson JE, Lasater B (2018). The imperative for patient‑centered clinical decision support. EGEMS (Wash DC).

[54] Krishnamurti L, Ross D, Sinha C (2019). Comparative effectiveness of a web-based patient decision aid for therapeutic options for sickle cell disease: randomized controlled trial. J Med Internet Res.

