# Supplementary material for: Machine-Learning Predictive Tool for the Individualized Prediction of Outcomes of Hematopoietic Cell Transplantation for Sickle Cell Disease: Registry-Based Study
Source: JMIR AI. 2025 Sep 15;4:e64519. doi: 10.2196/64519 (PMC12435087; doi:10.2196/64519)
Supplement: Multimedia Appendix 1 [file ai-v4-e64519-s001.docx]

| Supplementary Table 1: Feature Distribution of the Dataset | | |
| --- | --- | --- |
| Feature | Value | Count |
| AGEGPFF | <=10 | 718 |
| AGEGPFF | 11-17 | 487 |
| AGEGPFF | 18-29 | 309 |
| AGEGPFF | 30-49 | 114 |
| AGEGPFF | >=50 | 13 |
| SEX | Male | 889 |
| SEX | Female | 752 |
| GRAFTYPE | Bone marrow | 1137 |
| GRAFTYPE | Peripheral blood | 333 |
| GRAFTYPE | Umbilical cord blood | 171 |
| KPS | >=90 | 1226 |
| KPS | <90 | 256 |
| KPS | NaN | 159 |
| HCTCIGPF | 0-2 | 903 |
| HCTCIGPF | 3 | 475 |
| HCTCIGPF | NaN | 263 |
| DONORF | HLA identical sibling | 1010 |
| DONORF | HLA mismatch relative | 289 |
| DONORF | Mismatched unrelated donor and cord blood | 175 |
| DONORF | Matched unrelated donor | 167 |
| CONDGRPF | Myeloablative | 833 |
| CONDGRPF | Reduced-intensity conditioning | 378 |
| CONDGRPF | Non-myeloablative | 366 |
| CONDGRPF | NaN | 64 |
| CONDGRP_FINAL | Bu/Cy | 546 |
| CONDGRP_FINAL | Flu/Mel | 274 |
| CONDGRP_FINAL | Flu/Bu | 264 |
| CONDGRP_FINAL | TBI alone (300/400cGy) | 121 |
| CONDGRP_FINAL | Flu/Mel/TT | 114 |
| CONDGRP_FINAL | TBI/Cy/Flu | 110 |
| CONDGRP_FINAL | TBI/Cy/Flu/TT | 98 |
| CONDGRP_FINAL | NaN | 31 |
| CONDGRP_FINAL | TBI/Cy | 23 |
| CONDGRP_FINAL | Flu/Bu/TT | 21 |
| CONDGRP_FINAL | TBI/Flu | 13 |
| CONDGRP_FINAL | Cy/Flu | 9 |
| CONDGRP_FINAL | Treosulfan | 8 |
| CONDGRP_FINAL | TBI/Mel | 6 |
| CONDGRP_FINAL | Cy alone | 2 |
| CONDGRP_FINAL | Bu/Mel | 1 |
| ATGF | ATG | 780 |
| ATGF | Alemtuzumab | 700 |
| ATGF | None | 134 |
| ATGF | NaN | 27 |
| GVHD_FINAL | CNI + MTX | 710 |
| GVHD_FINAL | CNI + MMF | 308 |
| GVHD_FINAL | Post-CY + siro +/- MMF | 189 |
| GVHD_FINAL | Siro alone | 115 |
| GVHD_FINAL | CNI alone | 110 |
| GVHD_FINAL | CD 34 selection | 61 |
| GVHD_FINAL | Post-CY + MMF + CNI | 59 |
| GVHD_FINAL | NaN | 34 |
| GVHD_FINAL | Ex-vivo T-cell depletion | 25 |
| GVHD_FINAL | MTX alone | 12 |
| GVHD_FINAL | CNI + siro | 6 |
| GVHD_FINAL | MMF alone | 5 |
| GVHD_FINAL | MMF + siro | 4 |
| GVHD_FINAL | MTX + siro | 2 |
| GVHD_FINAL | MMF + MTX | 1 |
| HLA_FINAL | 8/8 | 1177 |
| HLA_FINAL | <=6/8 | 383 |
| HLA_FINAL | 7/8 | 80 |
| HLA_FINAL | NaN | 1 |
| RCMVPR | Positive | 765 |
| RCMVPR | Negative | 745 |
| RCMVPR | NaN | 131 |
| NACS2YR | NaN | 1063 |
| NACS2YR | 0.0 | 381 |
| NACS2YR | 1.0 | 110 |
| NACS2YR | 2.0 | 48 |
| NACS2YR | 3.0 | 25 |
| NACS2YR | 4.0 | 13 |
| NACS2YR | 6.0 | 1 |
| EXCHTFPR | NaN | 1291 |
| EXCHTFPR | Yes | 215 |
| EXCHTFPR | No | 135 |
| VOC2YPR | NaN | 903 |
| VOC2YPR | Yes | 460 |
| VOC2YPR | No | 278 |
| VOCFRQPR | NaN | 1000 |
| VOCFRQPR | < 3/yr | 445 |
| VOCFRQPR | >= 3/yr | 196 |
