# Supplementary material for: Machine-Learning Predictive Tool for the Individualized Prediction of Outcomes of Hematopoietic Cell Transplantation for Sickle Cell Disease: Registry-Based Study
Source: JMIR AI. 2025 Sep 15;4:e64519. doi: 10.2196/64519 (PMC12435087; doi:10.2196/64519)
Supplement: Multimedia Appendix 2 [file ai-v4-e64519-s002.docx]

| Supplementary Table 2: Outcome Distribution | | |
| --- | --- | --- |
| Outcome | Value | Count |
| EFS | No event | 1212 |
| EFS | Event happened | 404 |
| EFS | NaN | 25 |
| OS | Alive | 1489 |
| OS | Dead | 152 |
| GF | No | 1323 |
| GF | Yes | 288 |
| GF | NaN | 30 |
| AGVHD | No | 1280 |
| AGVHD | Yes | 339 |
| AGVHD | NaN | 22 |
| CGVHD | No | 1245 |
| CGVHD | Yes | 366 |
| CGVHD | NaN | 30 |
