# Supplementary figures and images for: Machine-Learning Predictive Tool for the Individualized Prediction of Outcomes of Hematopoietic Cell Transplantation for Sickle Cell Disease: Registry-Based Study
Source: JMIR AI. 2025 Sep 15;4:e64519. doi: 10.2196/64519 (PMC12435087; doi:10.2196/64519)

Acute Graft-vs-Host Disease

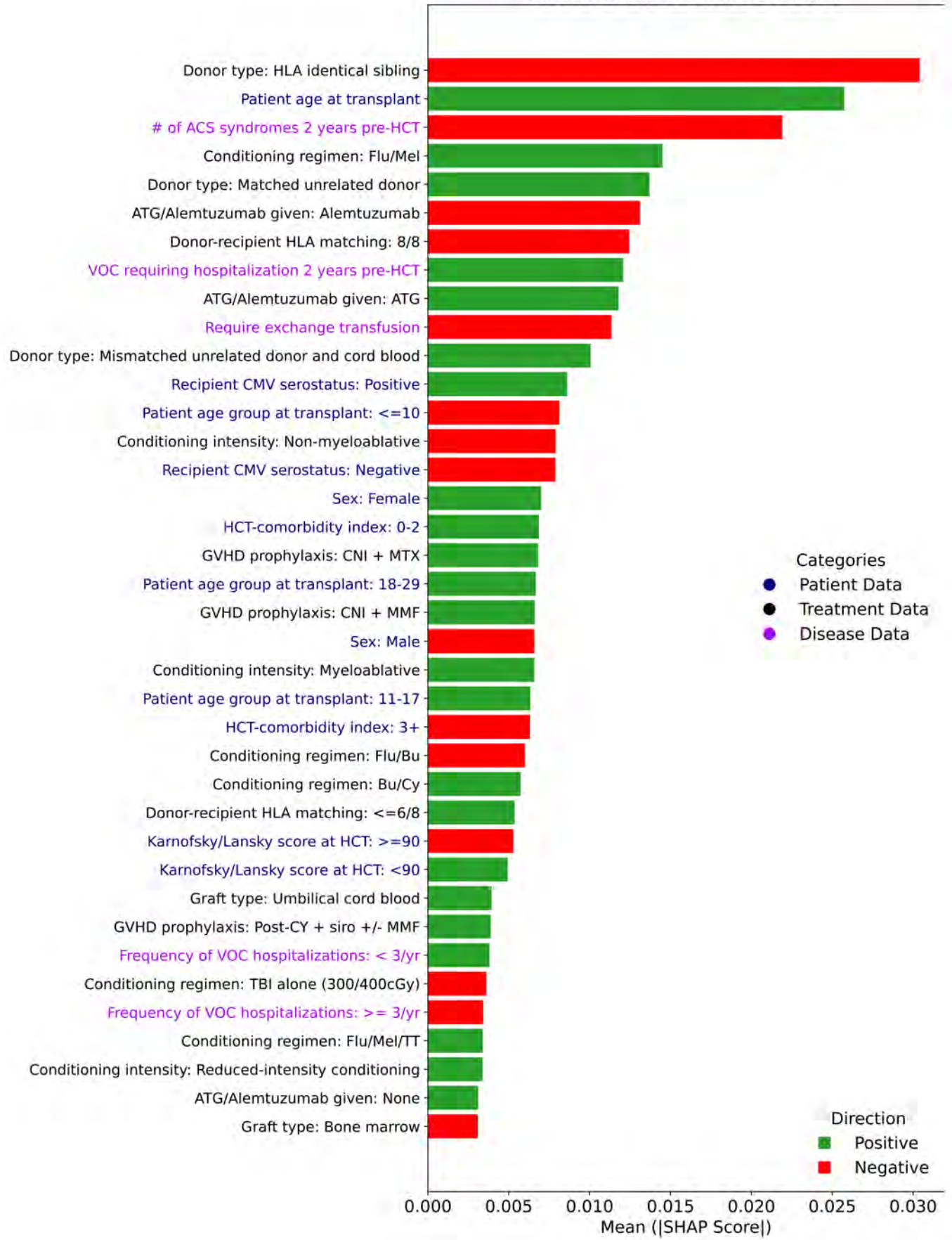

Supplement: Multimedia Appendix 3 [file ai-v4-e64519-s003.pdf]

# Graft Failure

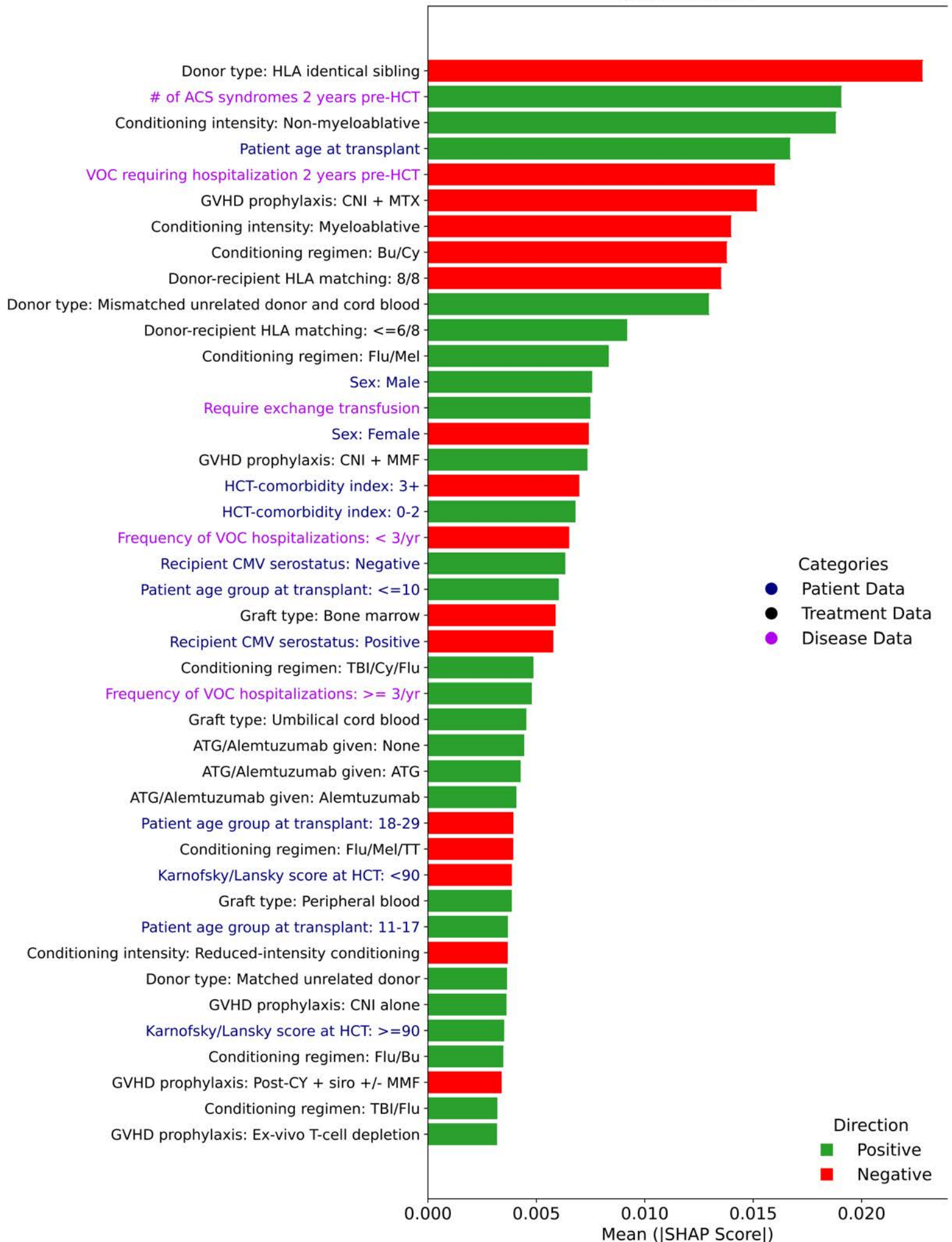

Supplement: Multimedia Appendix 4 [file ai-v4-e64519-s004.pdf]

# Event Free Survival

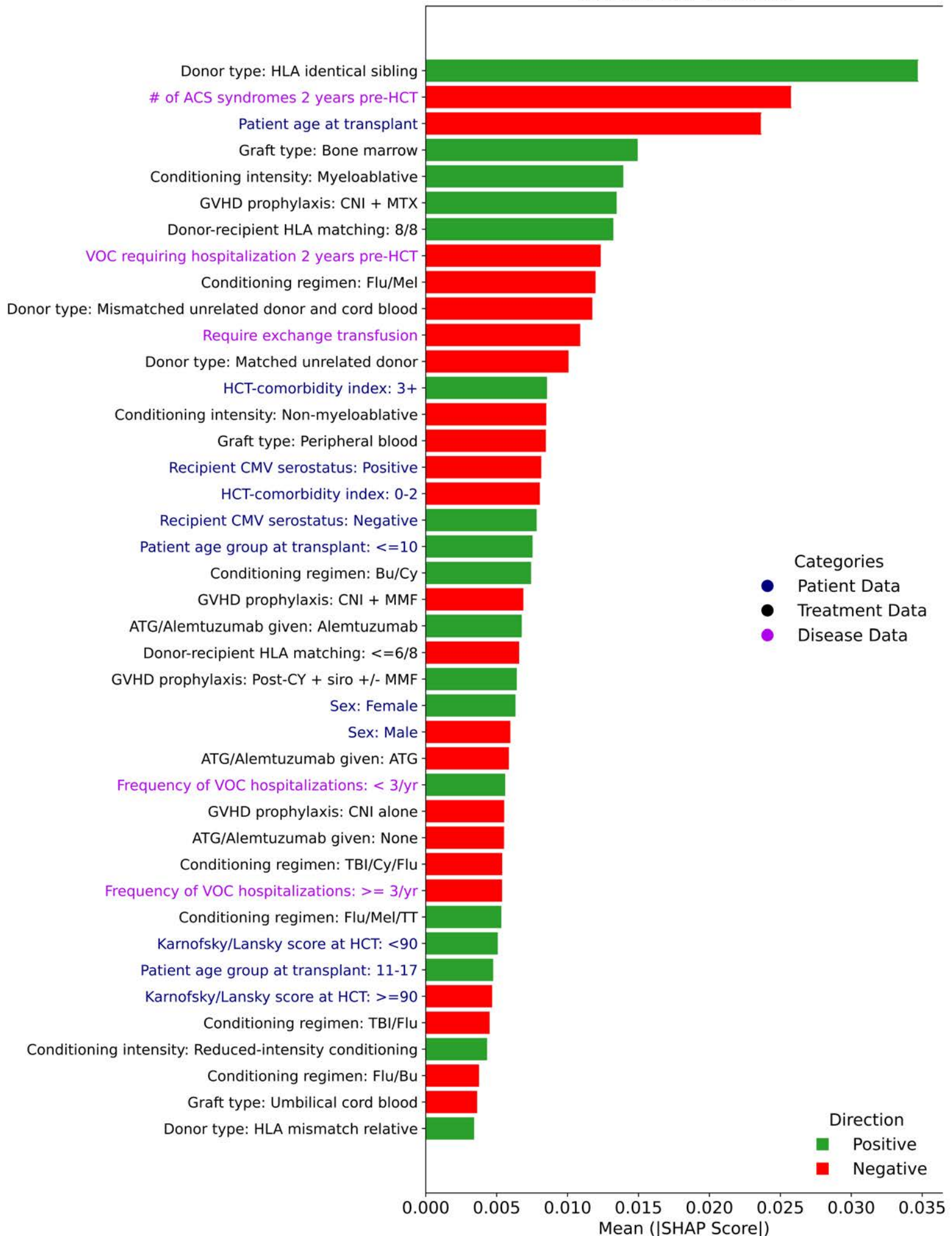

Supplement: Multimedia Appendix 5 [file ai-v4-e64519-s005.pdf]

# Chronic Graft-vs-Host Disease

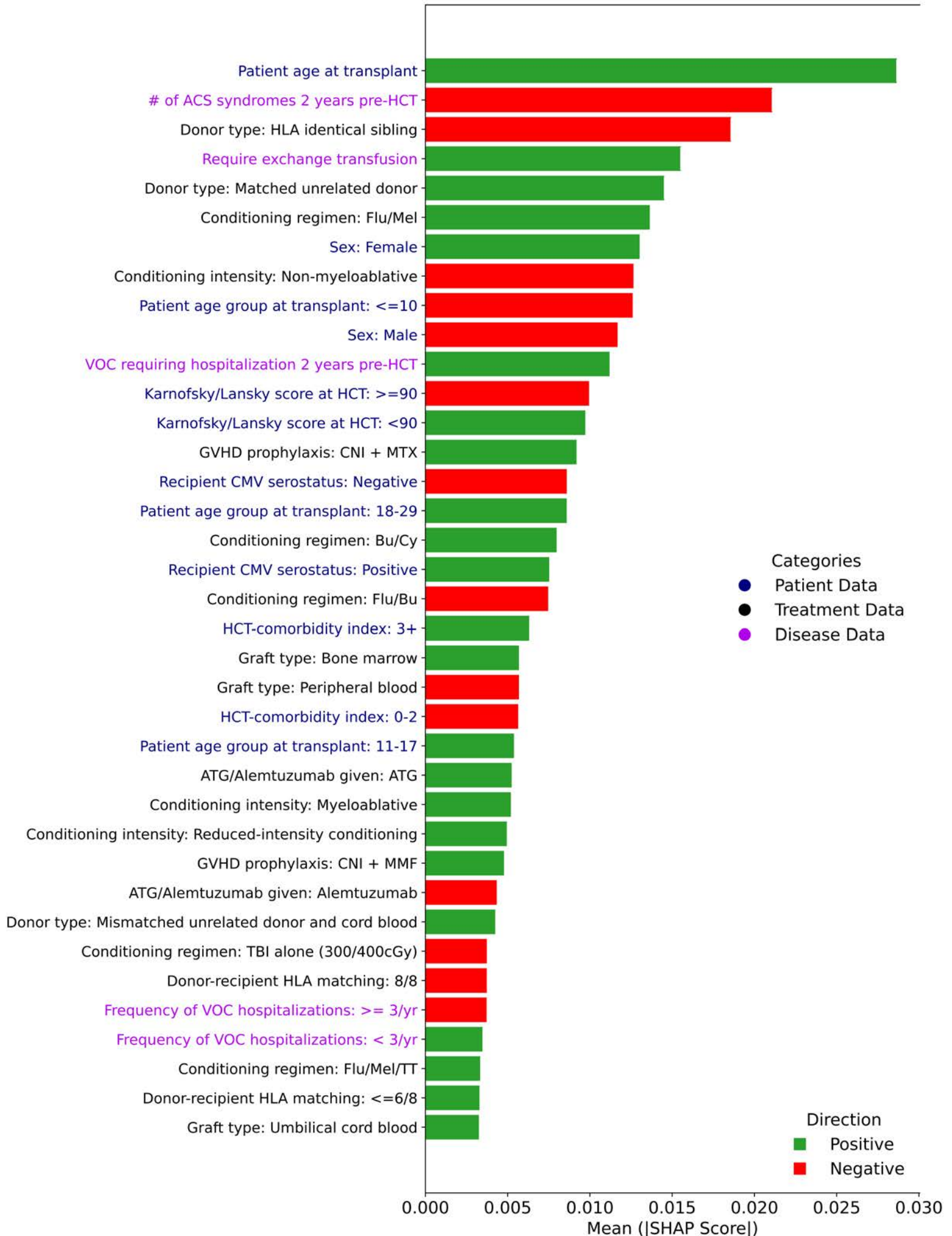

Supplement: Multimedia Appendix 6 [file ai-v4-e64519-s006.pdf]

Overall Survival

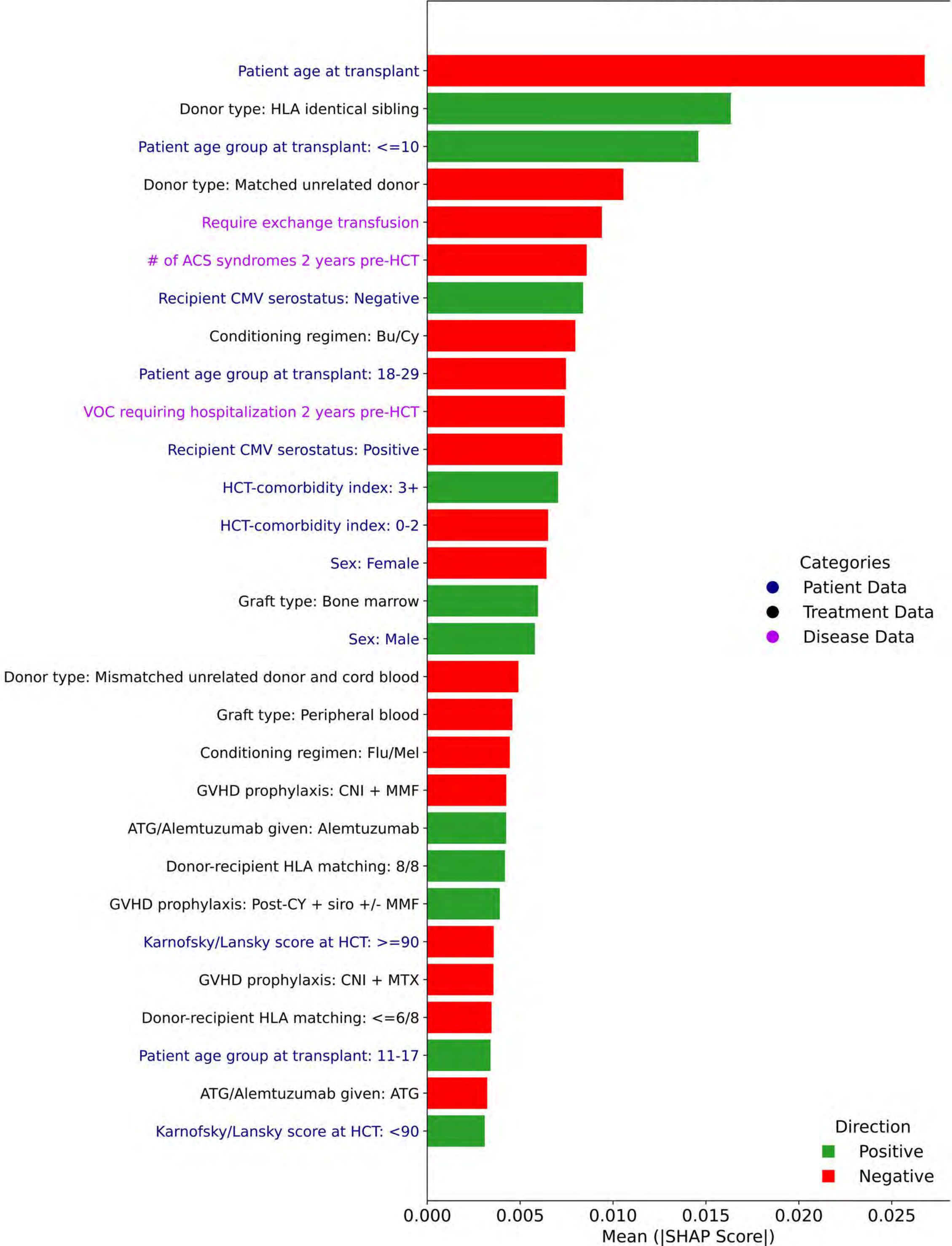

Supplement: Multimedia Appendix 7 [file ai-v4-e64519-s007.pdf]
