# Supplementary material for: Machine-Learning Predictive Tool for the Individualized Prediction of Outcomes of Hematopoietic Cell Transplantation for Sickle Cell Disease: Registry-Based Study
Source: JMIR AI. 2025 Sep 15;4:e64519. doi: 10.2196/64519 (PMC12435087; doi:10.2196/64519)
Supplement: Multimedia Appendix 8 [file ai-v4-e64519-s008.docx]

| Outcome | Patient 1 | Patient 2 | Patient 3 |
| --- | --- | --- | --- |
| EFS^a^ | 98.2 | 90.4 | 85.6 |
| OS^b^ | 98.8 | 92.9 | 90 |
| GF^c^ | 3.1 | 14.5 | 14.3 |
| AGVHD^d^ | 5.6 | 28 | 30.2 |
| CGVHD^e^ | 7.5 | 20.02 | 14.6 |

**Table 3.** Predicted probability percentage outcomes for each of the hypothetical patient profiles.

^a^EFS: event-free survival.

^b^OS: overall survival.

^c^GF: graft failure.

^d^AGVHD: acute graft-versus-host disease.

^e^CGVHD: chronic graft-versus-host disease.
