# Supplementary material for: Machine-Learning Predictive Tool for the Individualized Prediction of Outcomes of Hematopoietic Cell Transplantation for Sickle Cell Disease: Registry-Based Study
Source: JMIR AI. 2025 Sep 15;4:e64519. doi: 10.2196/64519 (PMC12435087; doi:10.2196/64519)
Supplement: Multimedia Appendix 9 [file ai-v4-e64519-s009.docx]

**Table 4.** Combinations of conditioning regimen, serotherapy, and graft-versus-host disease -prophylaxis used in published case series, which are included in the SPRIGHT.

| **Case series** |  | | | | |
| --- | --- | --- | --- | --- | --- |
|  | **Donor type** | **Conditioning regimen** | **Conditioning intensity** | **ATG^a^/alemtuzumab** | **GVHD^b^ prophylaxis** |
| Hsieh et al [7] | HLA^c^-identical sibling | TBI^d^ 300/400 cGy | Nonmyeloablative | ATG | Siro^e^ |
| King et al [10] | HLA-identical sibling | Flu/Mel^f^ | Reduced intensity | Alemtuzumab | CNI+MTX^g^ |
| Krishnamurti et al [8] | HLA-identical sibling | Flu/Bu^h^ | Myeloablative | ATG | CNI+MTX |
| Walters et al [3] | HLA-identical sibling | Bu/Cy^i^ | Myeloablative | ATG | CNI+MTX |
| Bolanos-Meade et al [42] | Mismatched relative | TBI/Cy/Flu^j^ | Nonmyeloablative | ATG/alemtuzumab | Post Cy+Siro+MMF^k^ |
| Patel et al [43] | Mismatched relative | TBI/Cy/TT/Flu^l^ | Nonmyeloablative | ATG | Post Cy+Siro+MMF |
| Shenoy et al [9] | Matched Unrelated | Flu/Mel | Nonmyeloablative | Alemtuzumab | CNI+MTX |
| Krishnamurti et al [8] | Matched Unrelated | Flu/Bu | Myeloablative | ATG | CNI+MTX |

^a^ATG: anti-thymocyte globuin.

^b^GVHD: graft-versus-host disease.

^c^HLA: human leukocyte antigen.

^d^TBI: total body irradiation.

^e^Siro:sirolimus.

^f^Flu/Mel: fludarabine+melphalan.

^g^CNI+MTX: calcineurin inhibitor+methotrexate.

^h^Flu/Bu: fludarabine +busulfan.

^i^Bu/Cy: busulfan+cyclosphosphamide:

^j^TBI/Cy/Flu: total body irradiation/cyclophsophamide/fludarabine.

^k^Post Cy+Siro+MMF: Post Cy+Siro+MMF: Post-HCT cyclophosphamide+sirolimus+mycophenolate moefetil.

^l^TT/Flu: total body irradiation+cyclphopshamide+thiotepa+fludarabine.
