# Supplementary material for: Machine-Learning Predictive Tool for the Individualized Prediction of Outcomes of Hematopoietic Cell Transplantation for Sickle Cell Disease: Registry-Based Study
Source: JMIR AI. 2025 Sep 15;4:e64519. doi: 10.2196/64519 (PMC12435087; doi:10.2196/64519)
Supplement: Checklist 1 [file ai-v4-e64519-s010.pdf]

Author Checklist Per Consolidated reporting guidelines for prognostic and diagnostic machine learning modeling studies

The following is the reporting checklist. A response indicates whether the particular item is documented in the study. Where the response to an item is Y then the location in the article is provided , and where the response is N or NA then the reasoning is provided.

#	Item	Y	N	NA	Location / Reasoning
**Study Details**					
1.1	*The medical/clinical task of interest*	✓			Introduction
1.2	*The research question*	✓			Introduction
1.3	*Current medical/clinical practice*	✓			Introduction
1.4	*The known predictors and confounders to what is being predicted / diagnosed*	✓			Introduction
1.5	*The overall study design*	✓			Introduction
1.6	*The medical institutional setting(s)*	✓			Methods-Dataset
1.7	*The target patient population*	✓			Introduction
1.8	*The intended use of the ML model*	✓			Introduction
1.9	*Existing model performance benchmarks for this task*	✓			Introduction
1.10	*Ethical and other regulatory approvals obtained*	✓			The institutional Review Board Yale University determined that this study did not constitute human Subjects research
**The Data**					
2.1	*Inclusion / exclusion criteria for the patient cohort*	✓			Methods- Dataset
2.2	*Methods of data collection*	✓			Methods - Dataset
2.3	*Bias introduced due to the method of data collection used*	✓			Discussion – Future Directions

2.4	*Data characteristics*	✓			Methods - Dataset
2.5	*Methods of data transformations and preprocessing applied*	✓			Methods - Dataset
2.6	*Known quality issues with the data*	✓			Discussion - Limitations
2.7	*Sample size calculation*	✓			Methods - Dataset
2.8	*Data Availability*	✓			Methods - Dataset
**Methodology**					
3.1	*Strategies for handling missing data*	✓			Methods - Dataset
3.2	*Strategies for addressing class imbalance*	✓			Methods – The Problem of Imbalance
3.3	*Strategies for reducing dimensionality of data*	✓			Methods – Feature Selection
3.4	*Strategies for handling outliers*			✓	No outlier detection done as working with CIMBTR patient data
3.5	*Strategies for data augmentation*			✓	No augmentation due to data being sourced from medical record
3.6	*Strategies for model pre-training*			✓	No pre-training done as only 1 source dataset and the ML model is a Random Forest
3.7	*The rationale for selecting the machine learning algorithm*	✓			Results - Evaluation Outcomes and Model Performance
3.8	*The method of evaluating model performance during training*	✓			Methods – Model Design
3.9	*The method used for hyperparameter tuning*	✓			Results – Evaluation Outcomes and Model Performance
3.10	*Model's output adjustments*	✓			Methods – Model Design
**Evaluation**					
4.1	*Performance metrics used to evaluate the model*	✓			Methods – Model Design
4.2	*The cost or consequence of errors*				Discussion - Limitations

4.3	*The results of internal validation*	√			Table 1
4.4	*The final model hyperparameters*	√			Table 5
4.5	*Model evaluation on an external dataset*		√		External Validation not done
4.6	*Characteristics relevant for detecting data shift and drift*		√		External Validation not done
**Explainability and Transparency**					
5.1	*The most important features and how they relate to the outcome(s)*	√			Results - Results - Evaluation Outcomes and Model Performance<br>Fig 5, Table 6,7
5.2	*Plausibility of model output*	√			Results - Results - Evaluation Outcomes and Model Performance
5.3	*Interpretation of model's results by an end-user*	√			Results – Spright User Interface
